# Prevalence and associated risk factors of Trypanosomosis in small ruminants of Abe Dongoro District, Western Ethiopia

**DOI:** 10.1016/j.parepi.2026.e00496

**Published:** 2026-03-21

**Authors:** Mitiku Wamile, Abdu Muhammed, Megersa Gemechu, Belay Beyene, Negasa Tamasgen

**Affiliations:** Faculty of Agriculture, Wollega University, Shambu, Ethiopia

**Keywords:** Trypanosomosis, Small ruminants, Prevalence, Associated risk factors, Abe Dongoro district, Ethiopia, Cross-sectional study

## Abstract

Trypanosomosis is a serious constraint to livestock productivity in Ethiopia, affecting a wide range of hosts. A cross-sectional study was conducted from October 2023 to December 2023 to determine the prevalence of trypanosomosis in small ruminants and its associated risk factors in the Abe Dongoro district, Oromia Regional State, western Ethiopia. A multi-stage sampling approach was employed for this study. A total of 390 blood samples (308 sheep and 82 goats) were collected from the ear vein using a random sampling method, considering various agro-ecological kebeles, body condition, age, and sex. The wet blood film examination and buffy coat technique were employed to determine the prevalence of trypanosomosis, and Giemsa-stained thin smears were used for species identification. Logistic regression analysis was used to assess associations between parasitic infection and risk factors. The overall prevalence of trypanosomosis in small ruminants was 17.69% (69/390), with 15.9% (49/308) in sheep and 24.39% (20/82) in goats. The dominant species of *Trypanosoma* identified were *T. congolense* (6.66%), *T. vivax* (4.87%), and *T. brucei* (1.02%), with mixed infections accounting for 5.13%. The difference in prevalence among parasite species was not statistically significant (*P* > 0.05). The study indicated that infection prevalence varied significantly across kebeles, body condition scores, and sex categories (*P* < 0.05). However, there was no statistically significant difference between age categories of animals (*P* > 0.05). The mean Packed Cell Volume (PCV) analysis recorded values of 20.76 ± 2.675 in parasitemic sheep and goats, compared to 26.78 ± 2.551 in aparasitemic individuals, revealing a significant statistical difference between the two groups (*P* < 0.05). The study concluded that trypanosomosis is prevalent in sheep and goats, with significant anemia and poor body condition in infected animals representing substantial productivity losses for smallholder farmers. The identification of kebele, sex, and body condition as independent risk factors enables targeted interventions. These findings underscore the urgent need for integrated control strategies combining vector control in high-risk areas, strategic trypanocidal drug use, and community education on animal husbandry practices to mitigate disease impact and improve livestock productivity in the region.

## Highlights

• Trypanosomosis prevalence is 17.69% in small ruminants in Abe Dongoro, Ethiopia.
• *T. congolense* is the dominant Trypanosoma species in infected animals.
• Parasite infection significantly lowers packed cell volume (PCV).
• Prevalence varies significantly with body condition and agroecology.

## 1 Introduction

Trypanosomosis is a complex protozoan disease caused by trypanosomes and primarily transmitted cyclically by tsetse flies. Trypanosomes are unicellular, flagellated protozoan parasites belonging to the genus *Trypanosoma* within the family Trypanosomatidae, order Kinetoplastida (Milgroom, 2023). These parasites are found in the blood and other tissues of vertebrates (Tesfaye and Ibrahim, 2017). In Africa, the Americas, and Asia, these diseases, which in some cases affect humans, result in significant illness in animals and cause major economic losses in livestock (Desquesnes et al., 2022). The disease results in significant economic losses and poses a major constraint on animal production in sub-Saharan Africa, particularly in Ethiopia, where it is widely distributed in areas where the vector, the tsetse fly (*Glossina* spp.), can survive (Getachew, 2005). Acyclical transmission of the parasite can also occur through biting flies of the Diptera order (such as Tabanus, Hematopota, and Stomoxys) (Baldacchino et al., 2013). Additionally, iatrogenic transmission can occur through the repeated use of contaminated vaccine syringes, treatment needles, or surgical instruments by animal health workers (Desquesnes and Dia, 2003).

According to Getachew et al. (2014), the most significant trypanosome species affecting livestock in Ethiopia include *T. congolense*, *T. vivax*, and *T. brucei* in cattle, sheep, and goats; *T. evansi* in camels; and *T. equiperdum* in horses, often resulting in mixed infections (Getachew et al., 2014). Khan et al. (2025) investigated *T.evansi* infection in the Balochistan province using the CATT/*T. evansi* kit method and de Barros Moura et al. (2024) confirmed the circulation of the *T. vivax* protozoan in cattle and buffalo herds in Amapá, predominantly in asymptomatic animals.

Trypanosomosis is a significant animal health issue that hinders settlement and socioeconomic progress in sub-Saharan Africa's tsetse fly belt regions. In Ethiopia, the disease is widespread, reported in multiple areas, and its incidence is rising. This poses a major challenge to livestock production and rural development (Cherenet et al., 2006; Habtamu, 2009; Molalegne Bitew et al., 2010). The rise in incidence can be attributed to several factors, including the effects of climate change (Sarwar et al., 2025; Ndiweni et al., 2025) on the transmission rate of the disease through vectors, the growing prevalence of the disease over time, and the decline in the number of trypanosomosis-resistant animal breeds as the overall population of susceptible animal increases. Small ruminants, such as sheep and goats, are recognized as important reservoir hosts for animal-infective trypanosome species in Ethiopia (Oche and Kasahun, 2025). Because these animals often exhibit greater trypanotolerance than cattle, they can maintain chronic infections and serve as a persistent source of transmission to other livestock within the same environment. While tsetse flies (Glossina spp.) are the primary cyclical vectors of trypanosomosis in Ethiopia, the epidemiological significance of mechanical transmission particularly for *Trypanosoma vivax* has been increasingly recognized (Fikru et al., 2016) and this mechanical transmission capacity allows *T. vivax* to persist in areas where tsetse control has been successful or in regions outside traditional tsetse belts. The Lake Tana Basin studies provide compelling evidence from Ethiopia, demonstrating active *T. vivax* transmission (6.1% prevalence in cattle) in the complete absence of tsetse flies, with abundant mechanical vectors including *Atylotus agrestis*(*Tabanidae*) and multiple *Stomoxys* species (Tegegne*,* 2004).

Small ruminants are particularly vulnerable due to their grazing habits and exposure to tsetse fly habitats (Hordofa and Haile*,* 2017). This study focuses on the Abe Dongoro District, where both tsetse flies and mechanical vectors are present. While mechanical transmission occurs, the disease is primarily driven by the cyclical transmission of tsetse fly populations. Currently, there is limited information on the prevalence and associated risk factors of small ruminant trypanosomosis in Abe Dongoro District. This knowledge gap is not unique to the study area. Across Ethiopia and other tsetse-endemic countries, limited information on small ruminant trypanosomosis has historically hindered evidence-based planning for integrated control measures. Research efforts have disproportionately emphasized bovine trypanosomosis, creating a critical blind spot in our understanding of disease ecology. Consequently, small ruminants have been systematically excluded from government-led tsetse and trypanosomosis control campaigns, an oversight with significant epidemiological consequences. Therefore, the objective of this study was to determine the prevalence of trypanosomosis in small ruminants and the associated risk factors in the Abe Dongoro District.

## 2 Materials and methods

### 2.1 Study area

**Fig. 1:**
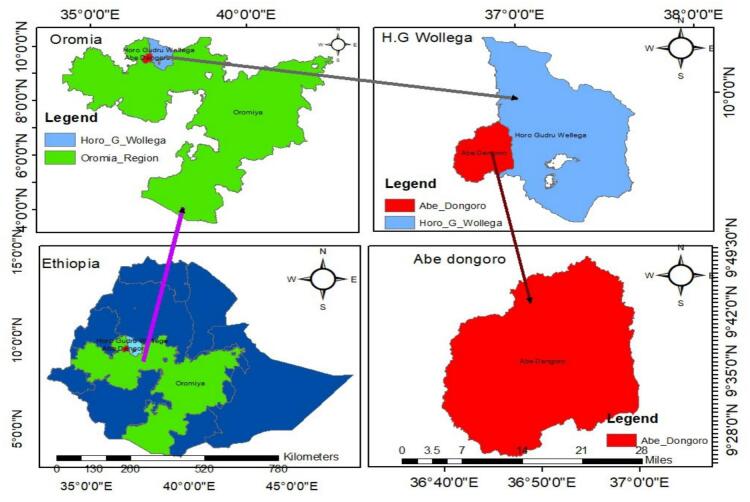
Map of showing study area.

The study was conducted in Abe Dongoro, located in the Oromia Regional State of Ethiopia. The district is situated 362 km from Addis Ababa, the capital city, and 47 km from Shambu town(zonal capital), as illustrated in Fig. 1. Abe Dongoro is geographically located at latitude 9°30′00“ N and longitude 36°49’59” E, at an elevation ranging from 1600 to 2300 m above sea level. Angar Gute (Gida Ayana district) borders Abe Dongoro to the west, Horro district to the east, Jardaga Jarte district to the north, and Gudaya Bila district to the south. The region experiences average temperatures between 12 °C and 32 °C, with annual rainfall ranging from 1750 to 2750 mm. The livestock population includes 92,050 cattle, 8092 sheep, 14,186 goats, 9803 equines, and 67,580 poultry (ADAO (Abe Dongoro Agricultural Office), 2022). Abe Dongoro encompasses three agro-ecological zones. Based on altitude, the land in the district is classified into three categories: highland, mid-highland, and lowland. The highland comprises 2.73% of the total land area, while the lowlands account for approximately 86.33%, and the mid-highlandcovers 10.94%. The temperature in Abe Dongoro is significantly influenced by altitude. The combination of the topography and other environmental features of the district has resulted in a diverse range of agro-ecological zones (ADAO (Abe Dongoro Agricultural Office), 2022).

#### 2.1.1 Study population

The study population comprised local breed sheep and goats of various age groups, body conditions, and sexes, all maintained under a traditional extensive husbandry system characterized by communal herding and grazing. Sheep and goats were considered eligible for inclusion in the study if they met all of the following criteria: (i) were indigenous sheep or goats born and raised in the study kebeles; (ii) were maintained under the traditional extensive management system typical of the area; (iii) were aged ≥6 months; (iv) no history of trypanocidal drug administration in the preceding 3 months (based on owner interview and treatment records) and (v) owner informed consent obtained. Animals were excluded if they met any of the following criteria: (i) had history of trypanocidal treatment within 3 months; (ii) had been introduced into the district from outside areas within the past 6 months; (iii) were in advanced pregnancy (third trimester); (v) signs of acute systemic illness unrelated to trypanosomosis; (vi) age < 6 months; or (vii) owner refusal to participate.

#### 2.1.2 Study design

A cross-sectional study was conducted from October 2023 to December 2023 to determine the prevalence of small ruminant trypanosomosis and to identify associated risk factors for the disease in three kebeles of the Abe Dongoro District. Various variables, including species, sex, age, and body condition scores, were recorded during sampling. The age of the animals was categorized as young (< 3 years) and adult (≥ 3 years) (Radostits et al., 2007). Additionally, the body condition of the animals was assessed and classified as good, medium, or poor based on the visibility of ribs and dorsal spines (Nicholson and Butterworth, 1986).

### 2.2 Sampling methods and sample size determination

A multi-stage sampling approach was conducted for this study. In the first stage, Abe Dongoro District was purposively selected based on its agro-ecological diversity, known presence of tsetse flies, and the absence of prior trypanosomosis research in small ruminants. From the district, three kebeles (Ido Kusa, Gorte, and Tulu Wayu) were purposively selected to represent the different agro-ecological zones present in the district. In the second stages, the sample size was calculated using the Thrusfield formula (Thrusfield, 2018).

$$n=\frac{Z^{2}\times P\left(1-P\right)}{d^{2}}$$

Where:

Z = statistic for 95% confidence interval (1.96).

P = expected prevalence.

n = required sample size.

d = desired absolute precision.

Due to the lack of prior information on prevelance of small ruminant trypanosomosis in the study district, an expected prevalence of 50% was used to maximize the sample size and ensure adequate statistical power. With a 95% confidence interval and a desired absolute precision of 5%, the calculated sample size was 384. This was increased to 390 to account for potential non-compliance or sample loss. To ensure representative sampling across species, proportional sampling was employed based on the relative population sizes of sheep and goats in the district. During the sampling period, sheep were more readily accessible in the selected kebeles due to seasonal grazing patterns and household distribution. Therefore, from the total sample of 390 animals, 308 were sheep and 82 were goats, reflecting the accessibility and proportional representation achievable during the study period.

In the final stage, within each kebele, households keeping sheep and goats were identified with the assistance of local development agents. From these households, individual animals were selected using a simple random sampling method. A sampling frame was created by listing all eligible animals (locally bred sheep and goats of both sexes and various ages) from participating households. Animals were then randomly selected. During sampling, stratification was applied to ensure representation across species (sheep and goats), sex (male and female), age categories (young <3 years and adult ≥3 years), and body condition scores (good, medium, poor), as these were the risk factors.

### 2.3 Sample collection

The area on the skin over the vein was cleaned with 70% alcohol and punctured using a sterile lancet. Two heparinized microhematocrit capillary tubes (75 mm length, 1.1–1.2 mm internal diameter) were filled to approximately 75% capacity with free-flowing blood from the ear vein puncture site. Each capillary tube holds approximately 50–60 μL of blood, yielding a total of 100–120 μL per animal for microhematocrit and buffy coat examination. One end of each tube was immediately sealed with cristaseal compound. An additional 1–2 mL of blood was collected directly into labeled 2 mL sterile plastic tubes containing EDTA (ethylenediaminetetraacetic acid) as anticoagulant. These tubes were gently inverted 5–6 times to ensure thorough mixing and prevent clot formation. Each capillary tube and EDTA tube was labeled immediately upon collection using a permanent marker. Following collection, all labeled samples were placed immediately in a portable cool box containing ice packs (maintaining temperature at approximately 4 °C) to preserve blood integrity and minimize parasite degradation. Samples were protected from direct sunlight and excessive heat during field collection. Samples were transported to the Abe Dongoro District Veterinary laboratory within 2–4 h of collection. Upon arrival, samples were processed immediately for parasitological examination. In cases where immediate processing was not possible, samples were temporarily stored in a refrigerator at 4 °C and examined within 12 h of collection.

### 2.4 Parasitological examination

Wet blood film, microhematocrit buffy-coat methods and Giemsa-stained thin blood smears parasitological examinations were conducted in the laboratory.

### 2.5 Wet blood film examination

To make a blood film, a drop of blood was put on a sterilized glass slide, and it was then carefully covered with a clean cover slip in order so that the blood spread uniformly. The slide was then placed under a microscope, and using an x40 objective lens, the film was methodically examined for the movement of the trypanosomes parasites.

### 2.6 Micro-hematocrit buffy coat microscopy

A blood sample was almost completely filled (approximately ¾) in a capillary tube with one end of the tube was sealed by crystal sealant, and it was centrifuged for five minutes at 12000 rpm. Then the PCV was measured and recorded for each sample. PCV levels below 26% was considered anemic for small ruminants (Radostits et al., 2007). The capillary tube was cut around 1 mm below the buffy coat layer to incorporate the topmost layer of red blood cells and 3 mm above it to incorporate some plasma. Using a micro-hematocrit capillary tube holder, the sample was carefully transferred onto a slide and then covered with a cover slip. After that, the preparation was evaluated under microscope using a phase contrast microscope to detect trypanosomes in the blood. The identification of the parasite species was conducted based on their movement in wet films and recorded for further confirmation by Giemsa-stained thin blood smear.

### 2.7 Giemsa-staining thin blood smears

The trypanosome species were identified by morphological differentiation of *Trypanosoma spp* based on size, shape, and location of the kinetoplast. *T. brucei* (slender, long free flagellum), *T. congolense* (smaller, no free flagellum) and *T. vivax* (large, free flagellum, prominent undulating membrane) were identified by observing under the microscope from Giemsa stained thin blood films prepared from the buffy coat of positive animals, which were examined using a microscope with a 100× oil immersion objective (Murray et al., 1997).Given that all three diagnostic methods employed were parasitological techniques based on direct visualization of the parasite, a hierarchical approach was adopted to define final infection status and to calculate prevalence. An animal was considered positive for *Trypanosoma* spp. infection if motile trypanosomes were observed in the wet blood film or if trypanosomes were visualized in the buffy coat layer extracted from the microhematocrit tube. The microhematocrit buffy-coat technique (MHCT) was treated as the primary screening tool due to its higher sensitivity compared to the wet film alone. To ensure specificity and allow for taxonomic classification, a positive result was only considered definitive for species-level prevalence if the parasite was observed and identified on the Giemsa-stained thin blood smear.

## 3 Data analysis

Data collected during the study were entered into Microsoft Excel (Microsoft Corporation, Redmond, WA, USA) and checked for completeness and consistency. The cleaned data were then exported to R statistical software version 4.3.3 (R Core Team, 2024) for analysis. Descriptive statistics were computed to summarize the prevalence of trypanosomosis across different variables (kebele, species, sex, age, and body condition). Prevalence was calculated as the proportion of positive animals divided by the total number examined, expressed as a percentage. For inferential statistics, both bivariate and multivariate analyses were performed. Initially, Pearson's chi-square (χ^2^) tests were used to assess associations between individual risk factors and trypanosome infection status. Multivariate logistic regression analysis was then employed to identify independent risk factors associated with trypanosome infection. A binary logistic regression model was constructed with trypanosome infection status as the dependent variable. Independent variables (risk factors) included in the initial model were: kebeles, sex, body condition, species and age. For continuous variables (PCV), an independent samples *t*-test was used to compare mean PCV values between parasitaemic and aparasitaemic animals. For all statistical analyses, the confidence level was set at 95%, and a significance threshold of *P* ≤ 0.05 was established.

## 4 Results

### 4.1 Parasitological findings

Among the 390 animals sampled to determine the prevalence of trypanosomosis, the overall prevalence rate was found to be 69 out of 390 (17.69%). This rate was 15.9% in sheep and 24.39% in goats, as shown in Table 1. The difference in the prevalence rates of trypanosomosis between sheep and goats was statistically significant (*P* = 0.006). Moreover, the prevalence rate of trypanosomosis in males (25.69%) was significantly higher than in females (13%) (*P* < 0.05). There was no significant difference in the prevalence of trypanosomosis across different age categories (*P* > 0.05). The prevalence of trypanosomosis among sheep and goats with poor, medium, and good body condition was 31.34%, 17.13%, and 6.94%, respectively. The differences in prevalence among these three body condition groups were statistically significant (P < 0.05). Additionally, there was a significant difference in the prevalence of trypanosomosis among the three kebeles (P < 0.05) and the study revealed various species of *Trypanosoma* in the study with statistical insignificance (Table 2).

**Table 1:** Prevalence of small ruminant Trypanosomosis and associated risk factors in relation to villages, species, sex, body condition and age of the animals in the study area.

Variables	Number of Examined animals	Number of Positive animals (%)	X^2^	*P*-value
**Villages(kebeles)**				
Ido kusa	110	11(10)	6.2786	0.04331⁎
Gorte	158	32(20.25)		
Tulu wayu	122	26(21.31)		
**Species**				
Sheep	308	49(15.90)	2.6429	0.104
Goat	82	20(24.39		
**Sex**				
Male	144	37(25.69)	9.1865	0.0024⁎
Female	246	32(13.0)		
**Age**				
< 3 years	139	20(14.38)	1.661	0.203
≥3 years	251	39(15.53)		
**Body condition**				
Thin	67	21(31.34)	14.34	0.00076⁎
Moderate	251	43(17.13)		
Good	72	5(6.94)		

⁎ Significant.

**Table 2:** The distribution of *Trypanosoma* species among various villages in the study area.

Village	No of Positive animals	*T.vivax*	*T. Congolense*	*T. brucie*	Mixed infection	X^2^cal	P-value
Idokusa	11	1	4	1	5		
Gorte	32	9	12	2	9	5.263	0.5105
T/wayu	26	9	10	1	6		
Infection rate (%)	–	4.87%	6.66%	1.02%	5.13%		

### 4.2 Hematological findings

The mean Packed Cell Volume (PCV) analysis was recorded as 20.76 ± 2.675 in parasitaemic sheep and goats, compared to 26.78 ± 2.551 in aparasitaemic individuals. The findings revealed a statistically significant difference (P < 0.05) between the two groups.

Multivariate logistic regression analysis was conducted to identify independent risk factors for trypanosome infection while controlling potential confounders (Table 3). The final model included kebele, sex, body condition, species, and age as predictors.

**Table 3:** Multivariate logistic regression analysis of prevalence of small ruminant Trypanosomosis and risk factors in the study area.

Risk factors	OR (95% CI)	P-value
**Sex**		
Female	Ref	Ref
Male	8.37(3.40–23.6)	0.001⁎
**Age**		
<3 years	Ref	Ref
≥3 years	1.22(0.52–2.88)	0.651
**Body condition**		
Good	Ref	Ref
Moderate	1.62(0.61–1.0)	0.366
Thin	65.9(13.5–531)	0.001⁎
**Village**		
Gorte	Ref	Ref
Tulu Wayu	0.61(0.21–1.64)	0.342
Ido kusa	0.009(0.001–0.043)	0.001⁎
**Species**		
Sheep	0.29(0.12–0.7)	0.006
Goats	Ref	Ref

⁎ Significant.

The analysis revealed that animals from Ido kusa kebele were more likely to be infected (OR = 0.009, 95% CI: 0.001–0.045, *P* = 0.001) and animals from Tulu Wayu kebele were more likely to be infected (OR = 0.61, 95% CI: 0.21–1.64, *P* = 0.342) compared to those from Ido Kusa kebele. Male animals had 8.37 times higher odds of infection compared to females (OR = 8.37, 95% CI: 3.40–23.6, P = 0.001). Body condition showed a strong association that animals in moderate body condition had 1.62 times higher odds (OR = 1.62, 95% CI: 0.61–1.0, *P* = 0.36), and animals in thin body condition had 65.9 times higher odds (OR = 65.9, 95% CI: 13.5–531, P = 0.001) of infection compared to those in good body condition. Goats showed a tendency toward higher infection risk compared to sheep, this did reach statistical significance (OR = 0.29, 95% CI: 0.12–0.0.7, *P* = 0.006). Age was not a significant predictor of infection status (*P* = 0.651).

## 5 Discussions

The overall prevalence of small ruminant trypanosomosis in the Abe Dongoro District was 17.69%. This could be associated with presence of vector (*Glossina spp*) in the study area and mechanical transmission of trypanosomes parasites. This finding is significantly higher than the 2.56% prevalence reported by Lelisa et al. (2016) in the Metekel Zone of the Benishangul Gumuz Region. Additionally, Tadesse and Megerssa (2010) documented a prevalence of 2.11% in small ruminants in the Guto Gida District of Western Ethiopia. Samdi et al. (2008) reported a prevalence of 2.10% at the Kaduna abattoir in Nigeria. Furthermore, the current finding exceeds the 10.57% prevalence reported by Mekonnen et al. (2014) in goats from the Abelti, Bede, and Ghibe valleys in other regions of Ethiopia. Human African Trypanosomiasis (HAT), also known as sleeping sickness, is a vector-borne parasitic neglected tropical disease (NTD) endemic in sub-Saharan Africa(Ortiz-Martínez et al., 2023).

The prevalence rate was assessed for different age categories: young animals (<3 years) and adults (>3 years), as age was considered a risk factor. In this study, the infection rate was higher in adult animals (15.53%) compared to young animals (14.38%), although this difference was statistically insignificant (*P* > 0.05). While not statistically significant, epidemiological studies often identify age as a risk factor due to biological mechanisms including cumulative exposure over time, age-specific grazing behaviors that influence vector contact rates, and waning of maternal immunity in young animals followed by gradual acquisition of partial immunity. The absence of association in this population may reflect uniformly high exposure risk across all age groups or limited statistical power to detect age effects. This finding is consistent with the study by Ayana et al. (2015). Additionally, the present study revealed that the infection rate increased with age. There was no statistically significant difference (P > 0.05) in the infection rates among different species of small ruminants (sheep and goats), which may be attributed to similar management systems and shared grazing areas.

The prevalence of trypanosomiasis in three kebeles was statistically significant (*P* < 0.05). This finding may be attributed to the study being conducted across different agro-ecological zones with varying climatic conditions. Among the three kebeles, Gorte kebele, classified as Kola in terms of agro-ecology, exhibited the highest prevalence rate of 20.25%. This elevated rate may be due to the relatively higher apparent density of tsetse flies, which is influenced by climatic factors and the vegetation coverage of the study area (Ayana et al., 2015).

The prevalence of trypanosomosis was observed among animals in poor, moderate, and good body condition, with infection rates of 31.34%, 17.13%, and 6.94%, respectively. These differences were statistically significant (P < 0.05). Trypanosomosis causes progressive emaciation through anemia, inflammation, and metabolic disruption, while poor nutritional status increases susceptibility through immune compromise. This finding contradicts previous reports from the Homosha district (Ayana et al., 2015) and the Goro district (Molalegne Bitew et al., 2010).

The strong association between body condition and trypanosome infection observed in this study warrants careful interpretation. Animals in thin body condition had 65.9 times higher odds of infection compared to those in good condition (OR = 65.9, 95% CI: 13.5–531, *P* = 0.001), representing the strongest risk factor identified. This relationship is likely bidirectional and reflects the complex pathophysiology of trypanosomosis. On one hand, chronic trypanosome infection causes progressive emaciation through multiple mechanisms: parasite-induced anorexia, increased metabolic demands due to fever and immune responses, cytokine-mediated catabolism, and anemia-induced tissue hypoxia leading to reduced nutrient utilization (Radostits et al., 2007; Taylor and Authié, 2004). On the other hand, animals with poor body condition may have compromised immune function due to protein-energy malnutrition, making them more susceptible to new infections or less able to control existing parasitaemia (Fekete and Kellems, 2007). Additionally, poor body condition may serve as a marker for other underlying health problems or increased exposure to vectors due to behavioral factors. The graded nature of the association (moderate condition: OR = 1.62; thin condition: OR = 65.9) suggests a dose-response relationship, further supporting the causal link between trypanosomosis and deterioration of body condition. This finding has important practical implications, as body condition scoring could serve as a simple, low-cost screening tool to identify animals at higher risk of infection in resource-limited settings.

There was a statistically significant difference in the mean Packed Cell Volume (PCV) between the parasitaemic group (20.76 ± 2.675) and the aparasitaemic group (26.78 ± 2.551) (*P* < 0.05). Trypanosome infection as a cause of anemia was evidenced by the decrease in packed cell volume of the infected animal. The mean PCV value recorded in this study was also significantly lower (P < 0.05) in parasitaemic (20.76%) than in aparasitaemic (26.78%) animals.

The study identified various species of *Trypanosomes*, with infection rates of *T. congolense* (6.66%), *T. vivax* (4.87%), and *T. brucei* (1.02%), as well as mixed infections (5.13%), which were found to be statistically insignificant (*P* > 0.05). This finding is consistent with the study conducted by Ayana et al. (2015). The present work aligns with the studies of Shimelis et al. (2011) and Fedasa et al. (2013), which reported that *T. congolense* was the predominant species and a major cause of cattle trypanosomiasis in the study area, followed by *T. vivax* and *T. brucei* (Mammo et al., 2013). A recent investigation into a different trypanosome species, *Trypanosoma melophagium*, in its specific vector *Melophagus ovinus* (the sheep ked) and reported T. melophagium infection from sheep in China for the first time(Huang et al., 2024). In contrast, the trypanosomes causing disease in livestock in our study area are likely transmitted by tsetse flies or other hematophagous dipterans with broader host ranges and different habitat preferences. Goats may exhibit different behaviors like browsing in higher-risk habitats or physiological traits that increase their exposure or susceptibility to these vectors and the specific trypanosome species they carry.

While the present study employed parasitological methods (wet blood film and buffy coat technique) for trypanosome detection, which are standard for field conditions in Ethiopia, other diagnostic approaches may yield different sensitivity profiles. Khan et al. (2025) utilized the CATT/*T. evansi* kit method in their investigation of *T. evansi* in Pakistan, a serological test that detects antibodies and can identify chronic or subclinical infections that might be missed by parasitological examination. The use of complementary diagnostic techniques in future studies could provide a more complete picture of trypanosome prevalence in the study area, particularly for detecting low-level parasitaemia or latent infections.

### 5.1 Limitation of the study

While this study provides valuable insights into the epidemiology of trypanosomosis in the study area, several methodological limitations should be considered when interpreting the findings. The cross-sectional nature of this study captures infection status and risk factor exposure at a single point in time, which precludes establishing temporal relationships between variables. Trypanosomosis diagnosis relied on conventional microscopic examination of stained blood smears, which has well-documented limitations in sensitivity. The study employed convenience sampling, selecting only three kebeles from the district based on accessibility and logistical considerations rather than random selection. This non-probability sampling approach introduces potential selection bias, as the selected kebeles may not be representative of all kebeles in the district. The study was conducted in only three kebeles within a single district, all characterized by similar farming practices. This restricted geographic scope substantially limits the generalizability of findings.

## 6 Conclusions

This study provides the first comprehensive evidence on the epidemiology of trypanosomosis in small ruminants in the Abe Dongoro District, revealing an overall prevalence of 17.69%. The study indicates the disease has statistically significant differences in susceptibility among kebeles, body conditions, and sex categories. These findings have important implications for targeted intervention strategies. The significant anemia observed in infected animals confirms the pathogenic impact of trypanosome infection on host physiology and explains the strong association with poor body condition. This represents a substantial economic burden for smallholder farmers, as affected animals have reduced productivity, growth rates, and market value. Based on these findings, we recommend implementing a comprehensive program combining vector control, strategic use of trypanocidal drugs, and community education on animal husbandry practices, with particular attention to the high-risk groups identified in this study.

## Consent to publish declaration

Not applicable.

## Consent to participate declaration

Not applicable.

## Clinical trial

Not applicable.

## CRediT authorship contribution statement

**Mitiku Wamile:** Writing – original draft, Methodology, Investigation, Formal analysis, Conceptualization. **Abdu Muhammed:** Writing – review & editing, Methodology, Investigation. **Megersa Gemechu:** Writing – review & editing, Visualization, Investigation. **Belay Beyene:** Writing – review & editing. **Negasa Tamasgen:** Writing – review & editing, Visualization, Formal analysis.

## Ethical approval

The study was conducted in accordance with ethical standards and has received approval from the Institutional Review Committee of Wollega University Shambu Campus (Reference No: WUSHC/IRC/23/065, dated September 11, 2023). All procedures involving animals were performed in compliance with relevant guidelines and regulations for the humane handling of livestock.

## Ethical consideration

Prior to the commencement of the study, the research proposal was reviewed and approved by the Research Ethics Review Committee of Wollega University. The committee evaluated all ethical aspects related to sample collection and handling. Sample collection was carried out by qualified and experienced professionals in accordance with approved ethical guidelines.

## Funding declaration

The authors declare that they did not receive any specific grants for this study from public, commercial, or not-for-profit funding agencies.

## Funding

Not received any specific grants for this study.

## Declaration of competing interest

The authors declare that none of the work described in this publication may have been influenced by any known conflicting financial interests or relationships.

## Data Availability

No data sets were generated and analyzed during the current study.
